# Evaluation of Nisin and LL-37 Antimicrobial Peptides as Tool to Preserve Articular Cartilage Healing in a Septic Environment

**DOI:** 10.3389/fbioe.2020.00561

**Published:** 2020-06-12

**Authors:** Ziba Najmi, Ajay Kumar, Alessandro C. Scalia, Andrea Cochis, Bojana Obradovic, Federico A. Grassi, Massimiliano Leigheb, Meriem Lamghari, Iraida Loinaz, Raquel Gracia, Lia Rimondini

**Affiliations:** ^1^Department of Health Sciences, University of Piemonte Orientale UPO, Novara, Italy; ^2^Center for Translational Research on Autoimmune and Allergic Diseases–CAAD, Novara, Italy; ^3^Faculty of Technology and Metallurgy, University of Belgrade, Belgrade, Serbia; ^4^Instituto de Engenharia Biomédica (INEB), Universidade do Porto, Porto, Portugal; ^5^Instituto de Investigação e Inovação em Saúde (i3S), Universidade do Porto, Porto, Portugal; ^6^CIDETEC Basque Research and Technology Alliance (BRTA), Donostia-San Sebastian, Spain

**Keywords:** antimicrobial peptides, pro-chondrogenic agents, co-cultures, bioreactor, human mesenchymal stem cells, antibacterial study

## Abstract

Cartilage repair still represents a challenge for clinicians and only few effective therapies are nowadays available. In fact, surgery is limited by the tissue poor self-healing capacity while the autologous transplantation is often forsaken due to the poor *in vitro* expansion capacity of chondrocytes. Biomaterials science offers a unique alternative based on the replacement of the injured tissue with an artificial tissue-mimicking scaffold. However, the implantation surgical practices and the scaffold itself can be a source of bacterial infection that currently represents the first reason of implants failure due to the increasing antibiotics resistance of pathogens. So, alternative antibacterial tools to prevent infections and consequent device removal are urgently required. In this work, the role of Nisin and LL-37 peptides has been investigated as alternative to antibiotics to their antimicrobial performances for direct application at the surgical site or as doping chemicals for devices aimed at articular cartilage repair. First, peptides cytocompatibility was investigated toward human mesenchymal stem cells to determine safe concentrations; then, the broad-range antibacterial activity was verified toward the Gram-positive *Staphylococcus aureus* and *Staphylococcus epidermidis* as well as the Gram-negative *Escherichia coli* and *Aggregatibacter actinomycetemcomitans* pathogens. The peptides selective antibacterial activity was verified by a cells-bacteria co-culture assay, while chondrogenesis was assayed to exclude any interference within the differentiation route to simulate the tissue repair. In the next phase, the experiments were repeated by moving from the cell monolayer model to 3D cartilage-like spheroids to revisit the peptides activity in a more physiologically relevant environment model. Finally, the spheroid model was applied in a perfusion bioreactor to simulate an infection in the presence of circulating peptides within a physiological environment. Results suggested that 75 μg/ml Nisin can be considered as a very promising candidate since it was shown to be more cytocompatible and potent against the investigated bacteria than LL-37 in all the tested models.

## Introduction

Articular cartilage (AC) represents a unique tissue in human body due to the lack of vascularization and innervation; it is composed of 80% water and populated by only one cell type represented by chondrocytes (Cohen et al., 1998). Despite its fundamental role in body support, AC exhibits a very low capacity for spontaneous repair mainly due to its avascular nature and a decreased mitotic capability of resident mature chondrocytes. These evidences represent a serious problem for clinicians as cartilage defects are not uncommon due to trauma (Aigner and Fan, 2003; Stone and Schaal, 2012; Makris et al., 2015), time deterioration and excessive body mass index (BMI) in both young and old population. As a further complication, AC defects are often associated with bone lesions and type II collagen breakdown (Widuchowski et al., 2007), thus causing pain and severe movement difficulties in affected patients.

Nowadays, treatment of focal chondral defects is mainly based on surgery and autologous chondrocyte transplantation. Surgery is performed onto the subchondral bone in order to create small microfractures and force stem cell migration from the neighboring bone to the cartilage injured site (Camp et al., 2014). Autologous chondrocyte transplantation includes collection and *in vitro* expansion of patient's own chondrocytes that are subsequently injected to repopulate the injured site (Camp et al., 2014). However, both treatment solutions are affected by severe limitations. In fact, autologous chondrocyte transplantation can result in terminal differentiation of the newly implanted cells into hypertrophy. Similarly, the microfracture surgery approach may lead to the formation of fibrocartilage, which is far less effective at bearing loads due to its poorer mechanical properties and weaker resistance to impact as compared to the native AC tissue. Moreover, the graft size and availability of cells for transplantation can be limiting factors despite good predicted outcomes of the selected therapy in certain cases. In addition, the surgical procedure can lead to bone damage (Chalmers et al., 2013). The problem of failures in cartilage repair procedures is probably underestimated: literature dealing with this surgery is not conclusive in identifying the causes of failure, and in many studies there is not even a clear definition of failure (clinical, radiological, or both).

Biomaterials science recently offered an alternative approach for articular cartilage repair based on the possibility to replace the injured tissue by an artificial scaffold substitute resembling the naïve tissue, thus allowing for temporary replacement and a progressive guided self-repair. Some promising examples include the use of hydrogels (Cochis et al., 2017; Cipriani et al., 2019; Bonifacio et al., 2020; Meng et al., 2020), polymers (Pourbashir et al., 2020; Xuan et al., 2020), and composites (Gao et al., 2019; Sun et al., 2019). However, the scaffold synthesis and surgical procedures aimed at the scaffold implantation open the possibility of bacterial infection. This is a rare eventuality (0.04–0.86%), but the clinical consequences can be particularly severe in case of septic arthritis (Stutz et al., 2000; Bauer et al., 2015; Wyatt et al., 2017). The improvements in biomaterials design for articular cartilage repair prefigure a larger use in implantology for the close future, with a potential increase in the number of septic complications.This evidence was recently reviewed by Bauer et al. which reported that the introduction of implants and transplants was probably the main reason of a 3-fold increase of infections (0.14–2.25%) in comparison with the data published previously (Bauer et al., 2015). Moreover, recent findings suggested also that low-grade infections might play a role, as it came to light recently for *Cutibacterium acnes* infection in shoulder surgery (Pruijn et al., 2020).

Articular cartilage infections have been commonly caused by *Staphylococcus aureus* and *Staphylococcus epidermidis* (Gram-positive), but other pathogens such as *Escherichia coli* (Gram-negative) can be responsible for septic arthritis, too (Balato et al., 2017). Once bacteria colonize the injured site, the infection evolves till a biofilm is formed, a 3D dense structure composed of proteins, lipids, DNA, and polysaccharides. In this scenario, the orthopedic surgeons' approach is based on the surgical removal of these biofilm communities; even in the case of a device implant, this represents a necessary pre-condition for eradication of the device-related infection. However, if the device infection proceeds to an advanced phase, the above-mentioned strategy is not sufficient. In fact, direct observation of the bacterial biofilm growth associated with orthopedic devices is very important for surgeons to determine the rational approach to the treatment; accordingly, when the bacterial biofilm is inherently resistant to the host defense and antibiotic therapy, both the device and the adherent biofilm have to be completely removed by a fastidious surgery in order to prevent the tissue infection. In fact, in the severe septic arthritis scenario, device removal and thorough debridement represent the only strategy to preserve surrounding tissues from the biofilm contamination. Accordingly, there is the urgent need for effective strategies to prevent septic arthritis by validating the use of new molecules effective in directly counteracting bacteria or suitable to be coupled with implantable biomaterials.

An interesting alternative to antibiotics is represented by antimicrobial peptides (AMPs). AMPs, or host defense peptides, were firstly discovered in the 1980's demonstrating effectiveness toward pathogenic bacteria, fungi and viruses (Sierra et al., 2017). They hold a double, direct and indirect activity toward bacteria. AMPs can directly bind to the negatively charged membrane phospholipids causing bacteria death due to pore formation, as well as by inhibitions of the ATPase actions of DnaK thus preventing chaperone-assisted protein folding (Rahnamaeian, 2011). In parallel, the presence of AMPs activates recruitment of neutrophils at the infection site, thus activating the immunological reaction cascade and indirectly counteracting the infection (Koziel and Potempa, 2013). Among the large class of AMPs, Nisin and LL-37 were considered of interest due to their proven antibacterial activity. Nisin is a lantibiotic widely used for preservation of food and beverages; it has been shown to be effective in counteracting mastitis (Fernández et al., 2008), dermatitis (Valenta et al., 1996) and gingivitis (Howell et al., 1993). LL-37 is the active cathelicidin peptide released by proteases from the pro-peptide hCAP18 transcribed from the human cathelicidin gene (Xhindoli et al., 2016). It has been shown to be beneficial for wound healing and skin protection by inhibiting proliferation of *Staphylococcus epidermidis* (Cogen et al., 2008).

So, considering the Nisin and LL-37 antibacterial activity, in this work their effectiveness in preserving articular cartilage healing in the presence of an infected environment was tested. Accordingly, AMPs *in vitro* ctytocompatibility was verified toward stem cell metabolism and chondrogenic differentiation in order to identify a range of safe concentrations. AMPs cytocompatible amounts were then investigated regarding antibacterial efficacy that is the ability in directly counteracting Gram-positive articular cartilage infection pathogens *S. aureus* and *S. epidermidis*; then, the AMPs broad range activity was further confirmed by using Gram-negative strains *E. coli* and *A. actinomycetemcomitans*, too. To test the AMPs selective antibacterial activity, a cells-bacteria co-culture assay was established. Next, a 3D cartilage-like spheroids model was applied to confirm the obtained results in a more physiologically relevant environment model; finally, a perfusion bioreactor was applied to simulate infection in the presence of circulating peptides within a physiological system.

## Materials and Methods

### Antimicrobial Peptides (AMPs)

Antimicrobial peptides (AMPs) were purchased from Merck (Merck, Milan, Italy). Sterile Nisin (liquid, ready-to-use solution, 1.02 g/ml in 0.02N HCl, SBR00021) and LL-37 (powder, human trifluoroacetate salt, 94261) were stored protected from light at 4°C and −20°C, respectively.

LL-37 mother solution was prepared by dissolving the powder into a 0.02N HCl solution (in sterile ultrapure water, from Millipore) until a final concentration of 1 mg/ml by vortexing (1 min, room temperature); all the procedures were performed under laminar air flow cabinet to preserve sterile conditions. AMPs solutions were prepared fresh prior to each experiment by diluting the AMPs' mother solutions directly in the appropriate cell or bacteria culture medium. AMPs were tested at the concentrations detailed in Table 1, based on previously reported MIC values in literature for LL-37 (Overhage et al., 2008; Leszczynska et al., 2013; Luo et al., 2017; Saporito et al., 2018; Kamysz et al., 2020) and Nisin (Brumfitt et al., 2002; Piper et al., 2009; Dosler and Gerceker, 2011; Iancu et al., 2012; Shin et al., 2015).

**Table 1:** AMPs tested concentrations (diluted in the appropriate medium).

**AMP**	**Tested Concentration (μg/ml)**	**Reported MIC range (μg/ml)**	**References**
Nisin	12.5, 25, 50, 75, 100	0.5–50	(Brumfitt et al., 2002; Piper et al., 2009; Dosler and Gerceker, 2011; Iancu et al., 2012; Shin et al., 2015)
LL-37	5, 7.5, 10, 25, 50, 75	1–50	(Overhage et al., 2008; Leszczynska et al., 2013; Luo et al., 2017; Saporito et al., 2018; Kamysz et al., 2020)

### Cytocompatibility

#### Cell Cultivation

Human bone marrow—derived stem cells (hMSC) were obtained from Merck (PromoCell C-12974) and cultivated in low-glucose Dulbecco's modified Eagle Medium (DMEM, Merck) supplemented with 15% fetal bovine serum (FBS, Merck) and 1% antibiotics (penicillin/streptomycin, Merck) at 37°C, 5% CO_2_ atmosphere. Cells were cultivated until 80–90% confluence, detached by a trypsin-EDTA solution (0.25% in PBS, from Merck), harvested and used for experiments.

#### Metabolic Activity Evaluation

Cells were directly seeded onto the wells of a 96 multiwell plate at a defined density (5x10^3^ cells/well) and cultivated for 24 h to allow adhesion and spread. Next, Nisin or LL-37 peptides were added to each well according to the concentration stated in Table 1 by directly diluting mother solutions into the medium and incubated for 24 h in direct contact with cells. Then, the cells viability was evaluated by means of metabolic activity by using the metabolic colorimetric Alamar blue assay (alamarBlue™, ready-to-use solution from Life Technologies, Milan, Italy) following the Manufacturer's instructions. Briefly, supernatants were removed from each well-containing cells and replaced with the Alamar blue solution (0.015% in fresh medium). Plates were incubated in the dark for 4 h at 37°C and then 100 μl aliquots were removed, placed into a new black 96 well plate and fluorescence signals were evaluated by a spectrophotometer (Spark, Tecan Trading AG, Basel, CH) using the following set-up: fluorescence excitation wavelength of 570 nm, fluorescence emission reading at 590 nm. Cells cultivated in the AMPs-free medium were considered as a control. The experiment was performed using six replicates for each application.

### Antibacterial Activity Evaluation

#### Strains Growth Conditions

Bacteria were purchased from the American Type Culture Collection (ATCC, Manassas, USA). To test the AMPs antibacterial activity, two Gram-positive *Staphylococcus aureus* (ATCC 43300) and *Staphylococcus epidermidis* (ATCC 14990), and two Gram-negative *Escherichia coli* (ATCC 25922) and *Aggregatibacter actinomycetemcomitans* (ATCC 33384) strains were used. Bacteria were cultivated on Trypticase Soy Agar (TSA, Merck) and incubated at 37°C until round single colonies were formed; then, 2–3 colonies were collected and spotted into 30 ml of Luria Bertani broth (LB, Merck, Milan, Italy). Broth cultures were incubated overnight at 37°C under agitation (120 rpm in an orbital shaker). A fresh culture was prepared prior to each experiment; bacteria concentration was adjusted to 1 × 10^5^ cells/ml by diluting in the fresh media until optical density of 0.001 at 600 nm was reached as determined by a spectrophotometer (Spark, Tecan, Mannedorf, Switzerland). Pure LB medium was used as a blank.

#### Biofilm Metabolic Activity Evaluation

Biofilms were formed under static conditions at the bottom of 96 well plates as previously described (Cochis et al., 2016). Briefly, 100 μl/well suspension containing 1 × 10^5^ bacteria prepared as described in the chapter Strains Growth Conditions was incubated for 90 min under agitation (120 rpm) at 37°C to force separation of the adherent biofilm cells and not-adherent floating planktonic cells (separation phase). Next, supernatants containing planktonic cells were removed and replaced with 200 μl of fresh media to cultivate surface-adhered biofilm cells (growth phase). Biofilms were grown at 37°C for 24 h. Afterwards, Nisin and LL-37 peptides were added to each well in direct contact with bacteria at the concentrations stated in Table 1 and incubated for 24 h at 37°C. The biofilm cells viability was assessed by means of metabolic activity by the Alamar blue assay as described above in the chapter Metabolic Activity Evaluation. Bacteria cultivated in the AMPs-free medium were considered as controls. The experiment was performed using six replicates for each application.

### Co-cultures

After cytocompatibility and antibacterial assays, the following optimal (intended as safe for cells and effective toward bacteria) AMPs concentrations were selected and used in further experiments: Nisin 75 μg/ml and LL-37 10 μg/ml. To verify the antibacterial activity of the targeted AMPs a cells-bacteria co-culture assay was performed as in our previous studies (Jekabsone et al., 2019; Cochis et al., 2020).

The co-culture method was designed to monitor the viability of both cells and bacteria that are challenging in the same environment for the same surface colonization, thus allowing for the validation of the AMPs' targeted activity toward bacteria (Cochis et al., 2020).

Briefly, cells (5 × 10^3^ cells/well) were seeded onto 96 well plates and allowed to adhere and spread for 24 h using the complete DMEM growth medium. The day after, medium was removed and replaced by a bacteria suspension prepared by using the antibiotic-free DMEM as described above for 24 h (DMEM was here used as blank for the optical density evaluation). Nisin (75 μg/ml) and LL-37 (10 μg/ml) peptides were added to test wells by diluting mother solutions directly in the medium while AMPs-free wells were used as controls. Results were collected after 24 h by means of viable cells counting by using trypan blue and bacteria number determination by the Colony forming unit (CFU) count. Briefly, after washing 3 times with PBS, cells and adherent bacteria were detached by 1 ml of trypsin-EDTA (0.25% in PBS) and collected into 1.5 ml tubes. Cells were immediately stained with trypan blue and counted using a Burker chamber. Viable bacteria numbers were obtained by the CFU count. Briefly, 100 μl of the bacteria/cell suspension were collected and transferred to a new 96 wells plate; here, 6 serials 1:10 dilutions were performed by mixing progressively 20 μl of the bacterial suspension with 180 μl of PBS as previously detailed in literature (Harrison et al., 2010). Then, 20 μl of each serial dilutions were spotted into a LB agar plate and incubated for 24 h until round colonies were visually checked; the final number of CFU was calculated by using the following formula (Harrison et al., 2010):

$$\begin{array}{l} CFU=\left[\left(number\;of\;colonies\right)\times {\left(dilution\;factor\right)}^{\left(serialdilution\right)}\right] \end{array}$$

The presence of cells in the control and infected wells were visually checked by light microscopy (Invitrogen EVOSfloid, Thermo Fisher Scientific, USA) prior to undergo cell and bacteria counts in order to provide a visual confirmation of the AMPs target activity. The experiment was performed using 3 different replicates for each application.

### Chondrogenesis

To exclude any interference due to the AMPs presence during cartilage repair, hMSC chondrogenesis was studied in an additional experimental series. Stem cells were seeded in 24 well plates at a density of 1x10^4^ cells/well for 24 h to allow adhesion and spreading. Then, 1 ml of the chondrogenic medium (DMEM High Glucose (4.5 g/L) supplemented with 10% ITS+1 Premix Tissue Culture Supplement, 10^−7^ M dexamethasone, 1 μM ascorbate-2-phosphate, 1% sodium pyruvate and 10 ng/mL transforming growth factor-beta 1 (TGF-β1), all from Merck, Milan, Italy) (Bonifacio et al., 2018, 2020) was added supplemented with 75 μg/ml of Nisin or 10 μg/ml of LL-37. The AMPs-free medium was considered as a control. Cells were cultivated for 21 days with the fresh medium change every 3 days. Finally, chondrogenesis was investigated by means of real-time PCR. Briefly, cells were digested for 5 min by Trizol (Merck, Milan, Italy) while RNA was reverse transcribed using the TaqMan reverse transcription kit. For real-time PCR, TaqMan Gene Expression Assay was used in a GeneAmp7500Real-Time PCR System (Applied Biosystems, USA). Data were normalized toward the housekeeping gene18S and compared to day 0 (=seeding day) by the ^ΔΔ^CT method (Cochis et al., 2017). Chondrogenic collagen-II (COLII), aggrecan (ACAN) and collagen-I (COLI) (all from Applied Biosystems, USA) expressions were evaluated. Finally, to confirm the gene expression results, cells were stained with specific chondrogenic markers Safranin-O and Alcian blue to confirm the presence of a cartilage-like matrix. Briefly, Safranin-O solution (1%) was prepared by diluting the commercial ready-to-use solution (Merck, Milan, Italy) in ultrapure water (MilliQ from Millipore), while Alcian Blue solution was prepared by dissolving 0,5 g in 50 ml of 3% acetic acid to a final pH of 2,5. Following the cell fixation in 4% paraformaldehyde (5 min, room temperature), dyes solutions were applied to cells for 15 and 30 min (room temperature) for Safranin-O and alcian blue, respectively. Finally, cells were washed three times with PBS to remove unbounded dyes and images were collected by light microscopy (Invitrogen EVOSfloid, Thermo Fisher Scientific, USA). Chondrogenesis experiments were performed using three replicates for each application.

### 3D Spheroids Studies

#### Spheroid Cultivation

To confirm the results obtained in monolayer cultures, a 3D cartilage-like spheroid model was realized according to the literature (De Hoogt et al., 2017; Langhans, 2018). Briefly, after detachment hMSC were counted and suspended in 15 ml tubes at a final concentration of 4x10^5^/ml using 1 ml of medium. The tubes were centrifuged (130 g, 5 min) to obtain a pellet; the hMSC pellet was maintained at the bottom of the tubes and cultivated in 1 ml of fresh medium. By this method, the pellet resembled a cartilage-like spheroid morphology after 48 h.

#### Spheroid Metabolic Activity Evaluation

To exclude any interference of the investigated AMPs with the 3D cartilage-like formation, spheroids were cultivated as detailed in the chapter Spheroid Cultivation and allowed to equilibrate for 48 h. Next, 1 ml of chondrogenic medium supplemented with 75 μg/ml of Nisin or 10 μg/ml LL-37 was added to the spheroids and cultivated in direct contact under static conditions for 48 h. Finally, the cell metabolic activity was verified by the Alamar blue assay; spheroids cultivated in the AMPs-free medium were considered as a control. Cell density in spheroids was visually inspected by confocal microscopy (Leica SP8 confocal platform, Leica Microsystems, Wetzlar, Germany): samples were fixed in formaldehyde (20 min, room temperature), permeabilized with Triton-X100 for 5 min at room temperature (0.1% in PBS, from ThermoFisher Scientific) and stained for 20 min with 4,6-diamidino-2-phenylindole (DAPI, Merck). After staining, the spheroids were gently laid down onto glass coverslips and immediately analyzed by the confocal microscope. The experiment was performed using 3 different samples for each application.

#### Spheroids-Bacteria Static Co-cultures

The AMPs targeted antimicrobial activity was investigated in co-cultures by using 3D spheroids and *S. aureus* as a reference strain for articular cartilage infections as we have previously shown (Bonifacio et al., 2018, 2020). Briefly, spheroids were allowed to equilibrate for 48 h in the complete medium; then, the medium was replaced by 1 ml of the antibiotic-free DMEM containing 1 × 10^5^ bacteria. Spheroids and bacteria were cultivated in direct contact for 48 h and then the numbers of viable cells and bacteria were determined. Specimens were collected into 15 ml tubes and enzymatically disaggregated by 1 ml of a collagenase/trypsin solution (1 mg/ml collagenase, 0.25% trypsin in PBS, all from Sigma-Aldrich) for 10 min at 37°C. Then, the tubes were vortexed for 3 times 30 s to favor cells separation and the trypan blue assay and the CFU count were used to evaluate the number of viable cells and bacteria, respectively. The experiment was carried using three different samples.

#### Chondrogenesis in Spheroids

As a further confirmation of the AMPs cytocompatibility, 3D spheroids were induced toward chondrogenesis as described above for cells in monolayers in the chapter Chondrogenesis. Accordingly, spheroids were allowed to equilibrate for 48 h in the basal medium (DMEM low glucose, 15% FBS) and then cultivated for 21 days in 1 ml/spheroid of the chondrogenic medium. AMPs (75 μg/ml Nisin or 10 μg/ml LL-37) were applied directly in the chondrogenic medium while AMPs-free spheroids were used as a control. Gene expression was determined after 21 days. Histology of spheroids was analyzed by means of the specific anti-collagen type II antibody (LS-C121638 from AbCam, UK); briefly, after fixing in formaldehyde (20 min, room temperature) the primary antibody (1:500 in a PBS 5% goat serum solution) was applied to the specimens and incubated overnight at 4°C. Then, the specimens were washed 3 times with PBS and collagen was unmasked by an appropriate secondary antibody (1:500, Alexa Fluor 568, ThermoFisher, USA); then, the cells were co-stained with 1:200 phalloidin (ab235137, AbCam, UK) and 1:1000 4,6-diamidino-2-phenylindole (DAPI, Merck, Milan, Italy) to visualize cytoskeleton f-actin filaments and nuclei, respectively. Fluorescence images were collected by a confocal microscope (Leica SP8 confocal platform, Leica Microsystems). The experiment was performed using 3 different samples for each application.

### Bioreactor Studies

After experiments under static conditions, a commercial 3D perfusion bioreactor (3D Perfuse, from the Innovation Center of the Faculty of Technology and Metallurgy, Belgrade, Serbia) was used to simulate 3D spheroids infection in a physiological environment. The bioreactor was equipped with a Shenchen LabN1 peristaltic pump (Baoding Shenchen Precision Pump Co. Ltd, China) and located in the incubator. Experiments were performed using the following conditions: 37°C, 5% CO_2_, 95% humidity, 300 μl/min flow rate (Freyria et al., 2005; Piola et al., 2013), 10 ml/bioreactor medium (DMEM low glucose, 15% FBS). The system was operated 24 h/day and the experiment was performed using three bioreactor cartridges with spheroids-bacteria co-cultures and Nisin and 3 bioreactor cartridges spheroids-bacteria co-cultures as controls.

### Spheroids-Bacteria Perfusion Bioreactor Co-cultures

After 48 h of cultivation in plates, spheroids were moved into the bioreactor chambers (1 spheroid/chamber); bioreactor was operated for 24 h to equilibrate the system at the conditions detailed in Bioreactor Studies. Then, medium was removed and replaced with an antibiotic-free DMEM supplemented with 1 × 10^5^ *S. aureus* from the inlet n.1 of the bioreactor (**Figure 9B**); immediately after, 75 μg/ml of Nisin was introduced in the system using the bioreactor inlet n.2 (**Figure 9B**) using 1 ml of DMEM medium. The AMPs-free medium containing bacteria was used as a control. The bioreactor was operated for 48 h to allow the complete interaction between the spheroids located in the chamber, the floating bacteria and Nisin. After this time, medium and spheroids were collected, enzymatically digested and analyzed for viable cell and bacteria counts by means of trypan blue and CFU, respectively, as described above in the chapter Co-cultures.

### Statistical Analysis of Data

Statistical analysis of data was performed using the Statistical Package for Social Sciences (SPSSv.20.0, IBM, US). Data normal distribution and homogeneity of variance was checked by Shapiro-Wilk's and the Levene's test respectively and then data were compared by ANOVA, followed by the Tukey's test. The significance level was set at *p* < *0.05*.

## Results and Discussion

### AMPs Cytocompatibility

The increase in septic arthritis cases due to the growing use of implants and transplants as well as due to the rise of pathogens antibiotic resistance urgently requires finding new candidates to counteract such infections as prior debated in the Introduction section. Antimicrobial peptides (AMPs) represent an interesting class of candidates due to their broad-range activity toward both Gram-positive and Gram-negative bacteria. This non-specific interaction between AMPs and bacteria is due to the AMPs structure: in general, they are short sequences of amino acids (ranging from 15 to 600) characteristic by the amphiphilic configuration, due to cationic residues on one side of the peptide, and hydrophobic residues on the other side (Hancock and Sahl, 2006). AMPs can be found in different districts of the human body such as skin, gut and saliva; they are mostly associated with inflammatory processes as they can be expressed by neutrophils and act as a natural killer after epithelium inflammation due to bacteria colonization as a defensive tool (Tjabringa et al., 2005). Accordingly, AMPs can be associated with toxic events and therefore their cytocompatibility has been assayed in this work first in order to determine Nisin and LL-37 safety concentrations prior to undergo with antibacterial properties evaluation. Human mesenchymal stem cells (hMSC) have been selected as model cells due to their pivotal role in tissue repair, while Nisin and LL-37 were assayed in a range of concentrations that were selected in accordance with their MICs in association with different strains previously reported in literature as detailed in Table 1. Therefore, our aim was to test whether these suggested amounts were compatible with stem cells in order to be applied as antimicrobial compounds in the context of a pathological state of articular cartilage undergoing healing.

**Figure 1:**
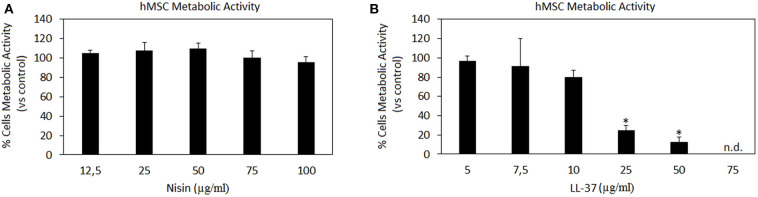
AMPs cytocompatibility. Nisin **(A)** was shown as *in vitro* cytocompatible at all the tested concentrations (*p* > 0.005 *vs*. the untreated control), while LL-37 **(B)** demonstrated high toxicity starting from the concentration of 25 μg/ml (*p* < 0.005 *vs*. the untreated control, indicated by the ^*^). Bars represent means and standard deviations. n.d., not detected.

Results are summarized in Figure 1. Nisin demonstrated high compatibility *in vitro*: negligible differences were detected in terms of hMSC metabolic activity between Nisin-doped and untreated control cells after 24 h of direct contact (Figure 1A, *p* > 0.05). These findings are in line with most of previous literature reports showing that Nisin is cytocompatible toward keratinocytes, fibroblasts and osteoblasts at concentrations <100 μg/ml (Shin et al., 2015). Still the use of Nisin in combination with stem cells with the aim of tissue repair was not studied yet. Here 100% metabolic activity was observed up to the Nisin concentration of 75 μg/ml, so that this concentration was selected as optimal for this AMP for further investigations.

Differently, LL-37 is known to be toxic at high concentrations due to its interactions with zwitterionic mammalian lipids (Lozeau et al., 2018); accordingly, concentrations <75 μg/ml were reported here (Figure 1B). Results confirmed previous findings, showing high LL-37 toxicity at concentrations above 25 μg/ml. In fact, cytotoxicity of LL-37 at the concentrations of 25 and 50 μg/ml was significant in comparison with the untreated control causing death of >70% cells (Figure 1B, *p* < 0.05 indicated by ^*^). Unlike for Nisin, some literature reports can be found regarding direct effects of LL-37 toward stem cells showing that the peptide was effective in promoting stem cell proliferation and differentiation: dental pulp stem cells (at 5–10 μg/ml LL-37 concentration, Milhan et al., 2017), adipose derived stem cells (at 2.5–20 μg/ml LL-37 concentration, Yang et al., 2016), and bone marrow derived stem cells (at 10 μg/ml LL-37 concentration, Yu et al., 2018). Still, in these works the applied LL-37 concentrations ranged between 5–20 μg/ml, thus confirming the possible use of this peptide only at low concentrations. So, in the present study the concentration of 10 μg/ml was considered as optimal for LL-37 for further investigations.

### AMPs Antibacterial Activity

After investigating the AMPs cytocompatibility *in vitro*, the same concentrations (with the exception of LL-37 at 75 μg/ml that was avoided due to the high toxicity) were applied toward 2 Gram-positive strains, *S. aureus* and *S. epidermidis*, as well as toward 2 Gram-negative bacteria that is *E. coli* and *A. actinomycetemcomitans*. Despite the evidence that *S. aureus* and *S. epidermidis* are mostly related with septic arthritis, the decision to extend the study toward two Gram-negative strains was made in order to evaluate any difference in efficacy of the selected AMPs. The main aim was to correlate these results with cytocompatibility results in order to determine Nisin and/or LL-37 concentrations that will ensure both safety and antimicrobial properties in potential applications.

**Figure 2:**
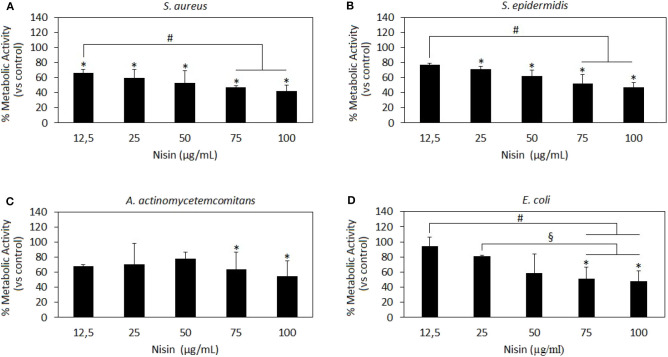
Nisin antibacterial activity. Nisin was shown as very effective toward the Gram-positive strains *S. aureus* **(A)** and *S. epidermidis* **(B)** wherein results at all concentrations >12.5 μg/ml are significant as compared to the untreated control (**A,B**, *p* < 0.05 indicated by the ^*^). Moreover, the results at 75 and 100 μg/ml concentrations were significant as compared to those at the lowest 12.5 μg/ml concentration (**A,B** *p* < 0.05 indicated by the ^#^), thus indicating a dose-dependent effect. Nisin 75 and 100 mg/ml showed also a high efficacy toward the Gram-negative *A. actinomycetemcomitans* **(C)** and *E. coli* **(D)** in comparison to untreated controls (*p* < 0.05 indicated by the ^*^) and lower concentrations (**D**, *p* < 0.05, indicated by the ^#^ and ^§^). Bars represent means and standard deviations.

**Figure 3:**
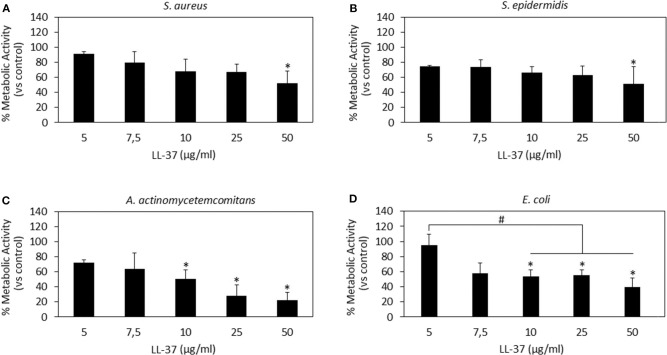
LL-37 antibacterial activity. LL-37 was shown as very effective toward the Gram-negative strains *A. actinomycetemcomitans* **(C)** and *E. coli* **(D)** at concentrations starting from 10 μg/ml inducing significantly different results as compared to the untreated controls (**C,D**, *p* < 0.05 indicated by the ^*^) and lower concentrations (**D**, *p* < 0.05 indicated by the ^#^). A less effective activity was noted toward the Gram-positive *S. aureus* **(A)** and *S. epidermidis* **(B)** where only the use of 50 μg/ml induced a significant reduction in the bacteria metabolism (*p* < 0.05 indicated by the ^*^). Bars represent means and standard deviations.

Results are reported in Figures 2 and 3 for Nisin and LL-37, respectively.

Nisin has shown to be more effective toward Gram-positive strains *S. aureus* and *S. epidermidis* (Figures 2A,B, respectively) as compared to Gram-negative strains (Figures 2C,D). In fact, all the concentrations >12.5 μg/ml resulted in significant reduction of the bacteria metabolism in comparison to untreated controls (Figures 2A,B, *p* < 0.05 indicated by the ^*^). Moreover, the increase in Nisin concentration up to 75 and 100 μg/ml induced a more evident metabolism reduction that was significant in comparison to the effects of the lowest concentration used of 12.5 μg/ml. Differently, only 75 and 100 μg/ml were effective to significantly reduce Gram-negative *A. actinomycetemcomitans* (Figure 2C, *p* < 0.05 indicated by ^*^) and *E. coli* metabolism (Figure 2D, *p* < 0.05 indicated by ^*^). Keeping in mind the primary goal to couple safety and antimicrobial properties, Nisin concentrations of 75 and 100 μg/ml look as promising candidates as they demonstrated high cytocompatibility and confirmed significant reduction in the viability of both Gram-positive and Gram-negative strains. These findings are in line with previous literature reports suggesting Nisin as an active agent toward Gram-positive bacteria. In fact, Nisin is classified as a Type A (I) lantibiotic (Asaduzzaman and Sonomoto, 2009), a class of molecules holding amphipathic and cationic properties known to affect Gram-positive bacteria (Smith and Hillman, 2008). The Nisin activity is targeted toward the integrity of the cellular membrane that is perturbed by forming small pores by binding the cell-wall precursor lipid II with high affinity (Wiedemann et al., 2001). This binding acts also as a precursor to inhibit other vital mechanisms such as cell wall biosynthesis, spore outgrowth, and activation of autolytic enzymes (Peschel and Sahl, 2006). This complex ensemble of different mechanisms is probably the reason why bacteria have difficulties in developing anti-Nisin strategies.

LL-37 results are reported in Figure 3. In general, only the 50 μg/ml concentration induced significantly different results as compared to those in the untreated controls for all the assayed strains (Figures 3A–D, *p* < 0.05 indicated by ^*^). In particular, LL-37 was found to be less effective toward the Gram-positive strains *S. aureus* (Figure 3A) and *S. epidermidis* (Figure 3B). However, this was not an encouraging finding as the 50 μg/ml concentration was shown as toxic toward hMSC in the cytocompatibility evaluation thus not displaying the required coupling of safety and antimicrobial properties. Moving to the Gram-negative strains, LL-37 was shown to be more effective; in fact, a statistically significant difference in comparison with the untreated controls was observed starting from the concentration of 10 μg/ml for both *A. actinomycetemcomitans* (Figure 3C, *p* < 0.05 indicated by ^*^) and *E. coli* (Figure 3D, *p* < 0.05 indicated by ^*^) where the same concentrations (10–25–50 μg/ml) induced significant different results also as compared to those obtained at the lowest concentration of 5 μg/ml (*p* < 0.05 indicated by #). Our results seem to be in line with those presented in literature where LL-37 was shown to exhibit a strong killing capacity toward Gram-negative strains such as *E. coli* whereas to only antagonize Gram-positive bacteria such as *S. aureus* (Wang et al., 2014). Similarly to Nisin, the difference can be ascribed to the non-targeted activity against the cell membrane: due to its cationic and amphipathic features, LL-37 can accumulate within bacteria cell walls leading to the disruption of the curved anionic membrane surfaces by development of pores (Wang, 2014). The latter are probably due to the ion channels that derive from the formation of long helix-bundle structures linking the AMP with the bacterial membrane that cause modification of the membrane permeability (Wang, 2014).

Finally, as a general consideration it should be mentioned that in this work the effect of AMPs was tested in a direct manner by using a metabolic assay to rank the peptides effects toward bacteria. So, further studies in the future should be addressed to verify other important aspects such as biofilm thickness that is a key step for drug resistance acquisition as well as the bacterial ability to develop intrinsic resistance toward the peptides. Moreover, the use of AMPs directly in the medium is probably not the best choice and it is open to some limitations related to leakage and targeting: ideally, surface modification and the use of nanocarriers will be considered in the future to minimize AMPs leaking and ensure it maintenance in the joint to avoid occurrence of septic arthritis. As an example, He et al. (2019) reported that LL-37 loaded onto silk fibroin nanoparticles enhanced recruitment and differentiation of stem cells toward bone-like phenotype onto titanium as well as macrophages activation. Similarly, Ron-Doitch et al. (2016) demonstrated how the toxicity of free LL-37 peptide was lowered by encapsulation within liposomes, thus confirming the importance of development of functional delivery systems to improve AMPs properties.

### Co-cultures

After the evaluation of AMPs cytocompatibility and antibacterial activity, the following concentrations were selected as the most promising in reducing the pathogen metabolism while preserving the cell viability that was considered as the key factor in this study: 75 μg/ml Nisin and 10 μg/ml LL-37. However, those results were obtained by keeping separated cells and bacteria in different environments; so, to check whether the AMPs activity was really targeting only (or at least mainly) bacteria, a co-culture assay was performed as we have previously described (Jekabsone et al., 2019; Cochis et al., 2020) to evaluate the AMPs performance in the same environment containing both cells and bacteria. Therefore, it should be clarified that only the number of adherent bacteria was evaluated as suggestive for those able to colonize the same surface as cells when cultivated in the same environment. This choice can represent the limitation of this technique as the lack of data related to floating viable bacteria can probably lead to an underestimation of the infection rate; however, this evaluation allows to focus on those bacteria that are able to adhere and proliferate onto a surface designed for cell population such as that of an implantable device thus representing the major risk for the healing success.

**Figure 4:**
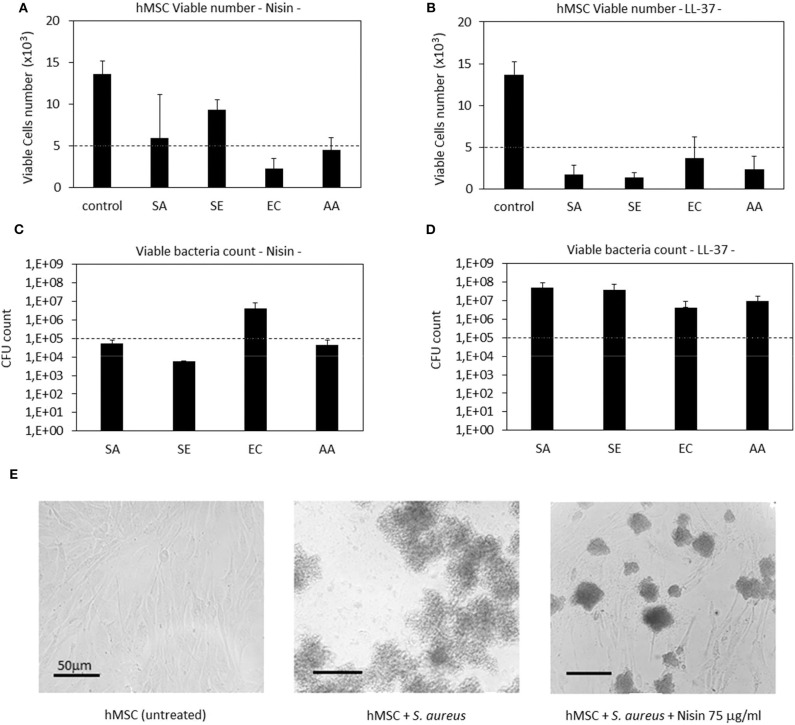
Cells-bacteria co-culture. Nisin showed a targeted activity in decreasing the viable bacteria number **(C)** and at the same time preserving cells **(A)**. Differently, the use of LL-37 was not effective in protecting cells from infection; in fact, they decreased from the starting number **(B)** while the bacteria were able to proliferate **(D)**. Finally, representative optical micrographs of *S. aureus* and hMSC cultures are shown **(E)** to demonstrate the difference between the control cells (left), cells and bacteria co-culture without Nisin (middle) and cells and bacteria co-culture with the addition of Nisin (right). Bars represent means and standard deviations; dashed lines indicate starting cell and bacteria numbers. SA, *S. aureus*; SE, *S. epidermidis*; EC, *E. coli*; AA, *A. actinomycetemcomitans*.

Results are reported in Figure 4. In general, Nisin demonstrated superior performances in comparison to LL-37. Looking at the viable cell count (Figure 4A), it was noticed that after 48 h cultivation (24 h seeding + 24 h in contact with the bacteria and AMPs) the presence of Nisin allowed the cell growth in the case of infection with *S. aureus* (SA) and *S. epidermidis* (SE). Conversely, the cell number was similar to the starting number (=seeding time) for the infection with *A. actinomycetemcomitans* (AA) whereas it was decreased when *E. coli* (EC) was used for the infection. These results were justified by the count of viable bacteria coming from the same environment as the cells (Figure 4C); in fact, the number of viable colonies was decreased in comparison to the starting number for SA, SE, and AA, while an increase of about 1 log was observed for EC. Coming back to the antibacterial assay results (Figure 2), a similar trend was here observed, thus confirming the Nisin strong activity toward Gram-positive strains. When cells were cultivated in the presence of bacteria without the AMPs addition, viable cells were not detected (data not shown). As a confirmation, representative images of cells infected with *S. aureus* in the presence of Nisin are shown (Figure 4E): while the infection induced the death of all cells (middle panel), the presence of 75 μg/ml of Nisin preserved most of the cells (right panel) that had a comparable morphology as the untreated controls (left panel). So, the targeted activity was verified for Nisin as it was able to counteract surface-adhered bacteria proliferation while preserving at the same time the ability of cells to grow.

LL-37 exhibited a lower antibacterial activity in comparison with Nisin when applied in the co-culture. In fact, all the strains applied in the assay induced a reduction in the cell number in comparison to the starting one (Figure 4B). As a consequence, the bacteria number was found to be increased in comparison to the starting infection (Figure 4D). So, the use of LL-37 for the co-culture did not satisfy the requirement to inhibit the bacterial proliferation for the benefit of cells as it was the case for Nisin. This difference can be probably ascribed to the different concentrations of applied AMPs (75 μg/ml Nisin vs. 10 μg/ml LL-37); in fact, it can be supposed that a part of the peptides aggregates with the medium proteins and precipitates thus losing its activity toward bacteria (Zozu et al., 2018). As debated above, direct use of AMPs in the medium is probably not the best solution for their administration due to leakage; surface modification and the use of nanocarriers can probably ameliorate AMPs effectiveness thus permitting reduction of the effective amount and allowing the use of more cytocompatible concentration ranges. However, according to the presented findings, the use of a low concentration of LL-37 was necessary due to its toxic effect toward stem cells as it was shown in the cytotoxicity assay (Figure 1).

### Chondrogenesis in Cell Monolayers

**Figure 5:**
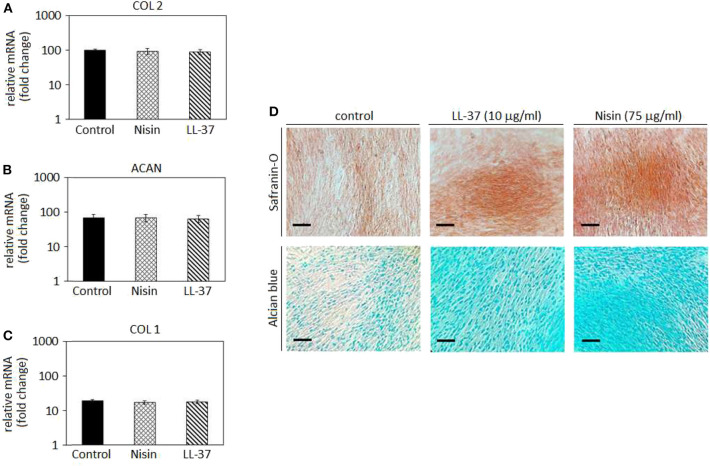
Chondrogenesis in cell monolayers. The use of 75 μg/ml Nisin or 10 μg/ml LL-37 did not affect the hMSC chondrogenesis as chondrogenic genes collagen type II (**A**, COL 2) and aggrecan (**B**, ACAN) were expressed in a similar manner as in the untreated control. As a confirmation, the osteogenic collagen type I (COL 1) gene was not up-regulated **(C)**. Histology **(D)** confirmed the presence of a cartilage-like matrix by specific Safranin-O and Alcian blue staining. Scale bar = 100 μm.

After confirming the AMPs cytocompatibility with mesenchymal stem cells, their role was investigated toward chondrogenesis. Stem cell commitment toward cartilage-like tissue in the presence of AMPs was assayed to exclude any possible interference of the peptides during the healing process. Accordingly, the selected AMPs concentrations (75 μg/ml Nisin and 10 μg/ml LL-37) were introduced into the chondrogenic medium to evaluate any differences in comparison to the AMPs-free controls. Results are reported in Figure 5.

In general, chondrogenic collagen type II (COL 2, Figure 5A) and aggrecan (ACAN, Figure 5B) expressions revealed that the presence of AMPs did not induce a down-regulation of these genes, thus suggesting that stem cell differentiation toward cartilage-like phenotype occurred in a similar manner as in the untreated controls. As a consequence, low expression of the bone-related collagen type I gene (COL 1, Figure 5C) confirmed that the cell differentiation moved toward the chondrogenic phenotype and not toward the osteogenic one. Such findings were predictable: as previously discussed in section AMPs Cytocompatibility, LL-37 was shown to be effective in promoting stem cells differentiation and adverse effects were not observed at the same concentrations (5–20 μg/ml) (Yang et al., 2016; Milhan et al., 2017; Yu et al., 2018). Conversely, at the best of our knowledge, Nisin was not assayed for effects on the stem cell commitment; however, the lack of toxic effects noticed in the cytocompatibility assay allowed for the speculation that Nisin could be also regarded as inert, not inducing any impairments to the stem cell commitment.

Gene expression results were visually confirmed by histology analysis (Figure 5D): in fact, cells cultivated with AMPs-doped medium were shown as positive for Safranin-O (upper panel) and Alcian blue (lower panel). These specific markers suggested the presence of glycosaminoglycans (GAGs) and proteoglycans in the cell matrix thus resembling a cartilage-like tissue. The lack of precipitates or a specific background suggests that AMPs did not interfere with the staining that can be therefore ascribed to the presence of GAGs and proteoglycans synthesized by the cells during cartilage commitment.

To the best of our knowledge, this is the first time that Nisin and LL-37 were investigated in relation to hMSC maturation toward cartilage. Considering the proved antibacterial and safety properties at the selected concentrations, our results suggest that the use of 75 μg/ml Nisin or 10 μg/ml LL-37 does not affect or interfere with hMSC maturation toward cartilage-like phenotype and represents a promising tool to preserve articular cartilage healing and protect from bacterial infection. However, a study from Baranska-Rybak et al. (2006) reported a decrease in the LL-37 antibacterial activity in biological fluids rich in GAGs; considering that the expression of GAGs represents the most indicative evidence of the cartilage-like commitment this evidence can represent a drawback for the use of LL-37 for cartilage healing thus pointing toward the use of Nisin.

### 3D Spheroid Studies

#### AMPs Cytocompatibility

**Figure 6:**
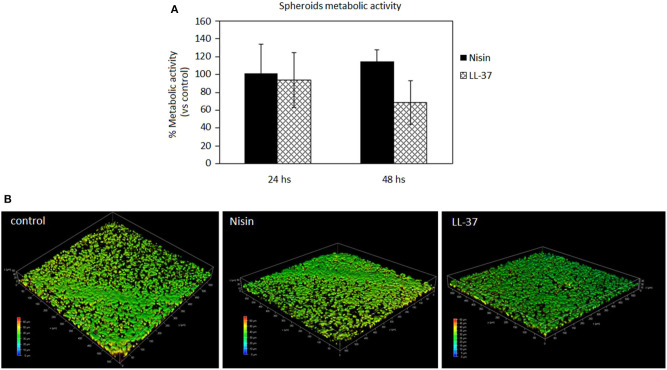
AMPs cytocompatibility in the spheroid 3D model. The use of both Nisin (75 μg/ml) or LL-37 (10 μg/ml) did not affect the cell metabolic activity (**A**, *p* > 0.05 *vs*. untreated controls) as well as the cell density **(B)**. Bars represent means and standard deviations.

After studies using cells in monolayers, a more physiological cartilage-like 3D spheroid model (Scalzone et al., 2019) was applied for further studies. This model is commonly applied for chondrogenic studies and is based on the use of mesenchymal stem cells that substitute chondrocytes for *in vitro* studies in order to overcome the poor expansion capacity of the latter cells. However, the key of this model is the cell-to-cell interaction that allows the formation of strong interconnections that are of a pivotal importance for chondrogenesis. Of course, the extracellular matrix is of fundamental importance to ensure tight connections of cells and the biochemical crosstalk. As previously debated, AMPs are able to link to the outer cell membrane thus representing a possible hitch for the spheroid integrity. Accordingly, after cell aggregation upon centrifugation, spheroids were cultivated for 24 and 48 h in a direct contact with AMPs; then the cell metabolic activity was evaluated and compared to that of the untreated spheroids that were considered as a control. Results are reported in Figure 6.

In general, the use of AMPs did not perturb the cell viability (Figure 6A, *p* > 0.05 *vs*. the untreated control) and the spheroid cells density, which was confirmed for all the specimens. These results were somehow expected as the applied AMPs concentrations (75 μg/ml Nisin and 10 μg/ml LL-37) were chosen according to their cytocompatibility. Cell density was not decreased as determined by observation of spheroids by using confocal microscopy and DAPI signals number evaluation (Figure 6B, representative for cell density recovered into the spheroids' core). By comparing results of 3D spheroids and cell monolayers, a similar tolerance toward AMPs was noticed. This is a promising result as the possible use of AMPs to preserve articular cartilage healing when infection occurs requires experimental investigations under physiological-like conditions to which are those in 3D spheroids much more similar than in the cell monolayers, especially for the matrix development (Scalzone et al., 2019).

It is again not straightforward to compare the obtained findings with literature reports as very few examples of the use of AMPs with 3D tissues (none dealing with cartilage-like tissues) can be found and none for Nisin. Lombardo Bedran et al. (2015) showed that LL-37 alone or in combination with other polyphenols was effective in reducing the inflammatory response in a three-dimensional co-culture model of gingival epithelial cells and fibroblasts. Accordingly, the present results encourage further investigations of the use of AMPs in 3D cell and tissue culture models.

#### Co-cultures

**Figure 7:**
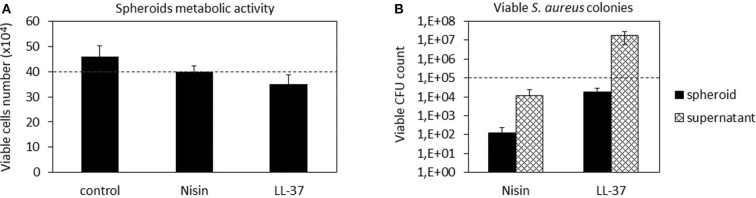
3D Spheroids-bacteria co-culture. The cell number **(A)** was preserved by both Nisin and LL-37 as values were comparable to the untreated control. Differently, Nisin confirmed a superior antibacterial activity in comparison to LL-37 as the number of viable bacteria in the supernatant was reduced for about 1 log **(B)**. Bars represent means and standard deviations; dashed lines indicate the starting cell and bacteria numbers.

After confirming the AMPs compatibility with 3D spheroids, the co-culture experiment was repeated to further confirm their targeted activity toward bacteria. Accordingly, as performed with cells in monolayers, the spheroids were allowed to equilibrate for 48 h in the complete medium and then they were infected with the antibiotics-free medium containing 1 × 10^5^ *S. aureus* and the peptides. After 48 h, the spheroids were enzymatically disaggregated and viable cells and adhered bacteria were counted by the trypan blue staining and CFU counts, respectively. Results are reported in Figure 7.

In general, we observed a lower decrease in the viable cell number for both AMPs after infection in comparison to the previous experiment performed with cells in monolayers (Figure 4). First explanation of these findings can be related to the hMSC intrinsic antibacterial properties (Mezey and Nemeth, 2015). In fact, while for the monolayer experiments 5 × 10^3^ cells were used, here each spheroid was composed of 4 × 10^5^ cells, thus holding ≈80× more cells. Furthermore, the cells were arranged in a 3D structure resembling the physiological environment, enhancing cell differentiation, and protecting the cells in the interior from the external factors. So, the number of cells in the spheroids was similar in all cultures and not significantly different from the initial number (Figure 7A). On the other hand, differences were noticed between the numbers of colonies adhered to the spheroids and the floating ones (Figure 7B). In fact, only few colonies were able to adhere to the spheroid while most of bacteria remained in the supernatant; here, the activity of Nisin was confirmed to be superior in comparison to LL-37 as it reduced the bacterial count for about 1 log in comparison to the starting number (Figure 7B). Accordingly, the message of the results obtained in the monolayer experiments were here confirmed, suggesting Nisin as the most promising candidate.

As a general consideration, in our opinion these findings also suggest the importance to perform analyses by using physiologically relevant models such as 3D cell and tissue cultures even for *in vitro* studies. This consideration seems to be of particular importance when cartilage is under consideration: in fact, as previously demonstrated for example by Kim et al. (2003) there is an evident difference in terms of the cell biological response in 2D monolayer and 3D tissue-like cultivation. Therefore, it can be supposed that a different cell response to the environmental changes (such as infection or injury) is also conditioned by the cultivation method applied for *in vitro* studies; in fact, in this study the performance of LL-37 was improved in 3D spheroid cultures in comparison with cell monolayers. Therefore, we believe that an important confirmation coming from this work is related to the appropriate choice of the study design as it is also evidenced in the fluidic studies described in chapter Bioreactor Studies.

#### Chondrogenesis in Spheroids

**Figure 8:**
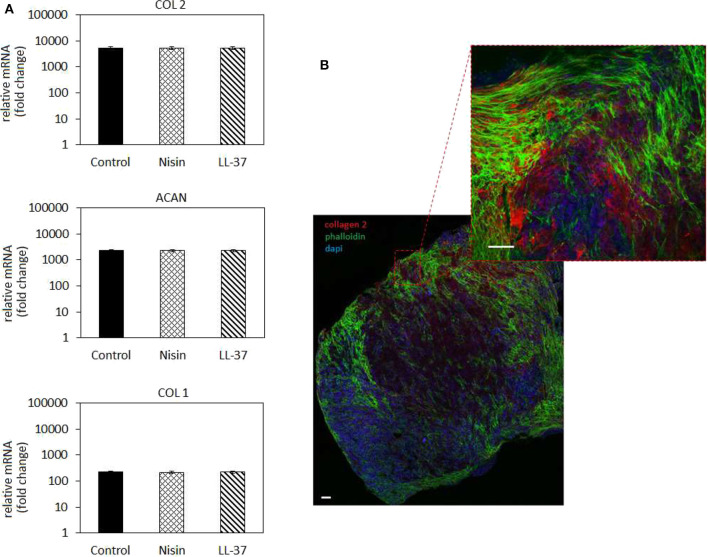
Chondrogenesis in 3D spheroids. **(A)** The use of 75 μg/ml Nisin or 10 μg/ml LL-37 did not affect the hMSC chondrogenesis: chondrogenic genes for collagen type II (COL 2) and aggrecan (ACAN) were up-regulated at the same levels as in the untreated control. Fluorescent images **(B)** confirmed the presence of a cartilage-like matrix by the presence of the specific collagen type II staining (in red). Scale bar = 100 μm.

The 3D spheroid model was further applied to study the hMSC chondrogenesis in the presence of AMPs. The use of high-density cell spheroids as a study model was indicated to resemble better the physiological conditions required for autologous cartilage repair in comparison to typical monolayer cultivation (De Hoogt et al., 2017; Langhans, 2018). Results are reported in Figure 8.

In general, chondrogenic genes collagen type II (COL 2) and aggrecan (ACAN) up-regulation revealed that the presence of AMPs did not interfere with stem cell commitment toward the cartilage-like phenotype as also observed for the cells in monolayers (Figure 5). Accordingly, low expression of the bone-related collagen type I gene (COL 1) provided a further proof that cells differentiated toward the chondrogenic phenotype and not toward the osteogenic one. However, by applying the 3D model a general more evident upregulation was observed for chondrogenic genes in comparison to the 2D model; in fact, COL2 as well as ACAN were much more upregulated in 3D culture (Figure 8) than in cells in monolayers (Figure 5). This was a further confirmation of the importance to apply 3D models also for *in vitro* studies to better mimic physiological conditions that influence cell responses.

The gene expression results were confirmed by fluorescent staining (Figure 8B, representative for Nisin): in fact, spheroids cultivated with AMPs-doped medium preserved their high cellular density (nuclei stained in blue by DAPI) as well as high cells-to-cell tight junctions were maintained as shown by the interconnected cytoskeletons (marked in green with phalloidin). Moreover, the strong red signal representative for chondrogenic collagen type II showed that the ECM formed within the spheroids is highly cartilage-like.

As discussed above, to the best of our knowledge this is the first manuscript hypothesizing the use of Nisin and LL-37 for articular cartilage healing preservation in a septic environment; accordingly, other works are not useful to compare with our findings. However, due to the lack of toxicity of the selected concentrations toward 3D spheroids and the expression of chondrogenic genes after the stem cell commitment in the presence of AMPs, we consider these findings as very promising to explore the possible use of Nisin and LL-37 for applications aimed at cartilage healing.

### Bioreactor Studies

**Figure 9:**
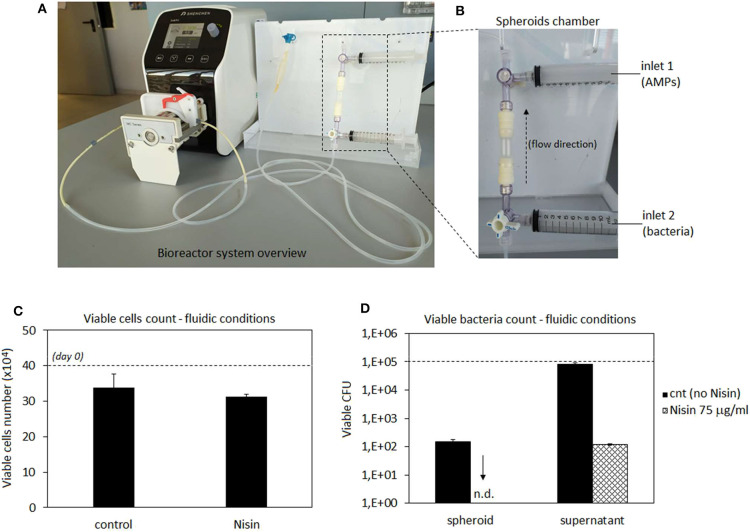
Bioreactor studies. A general overview of the perfusion bioreactor **(A)** and its chamber with 2 external inlets **(B)**. Nisin was very effective to counteract the *S. aureus* infection as the number of viable cells was preserved and comparable to that in the untreated control **(C)** while adherent bacteria were not detected on the spheroid surfaces and the number of floating bacteria was significantly reduced **(D)** by the presence of Nisin in comparison with the untreated controls. Bars represent means and standard deviations. n.d, not detected; dashed lines represent seeding number of cells and bacteria.

Taking into account cytocompatibility and antibacterial results obtained from both monolayer and 3D experiments, Nisin was considered as the more promising peptide for further evaluations. Accordingly, we moved from static to hydrodynamic conditions to better mimic the physiological environment. Articular cartilage is normally subjected to dynamic compression and shear deformations as the main mechanical stimuli, while inducing, in the same time, interstitial fluid flow. It has been recognized that fluid flow and corresponding shear stresses affect cell metabolism, differentiation, morphoregulation and ECM synthesis (Rutkowski and Swartz, 2006) although exact mechanisms are not yet precisely defined. Perfusion was shown to stimulate chondrocyte proliferation and ECM synthesis (Pazzano et al., 2000; Freyria et al., 2005; Chen et al., 2012) as well as chondrogenic differentiation of hMSCs (Alves da Silva et al., 2011) while the effects may be donor-dependent (Kock et al., 2014). In the case of *in vitro* cultivation of 3D cell and tissue cultures, such as spheroids ~2 mm in size in the present work, application of fluid flow is necessary to ensure efficient mass transport in the liquid phase and adequate delivery of both, bacteria and AMPs to the spheroids. Thus, we have used a perfusion bioreactor: the spheroids were hosted in the chamber while bacteria (*S. aureus*) and AMPs (Nisin 75 μg/ml) were separately introduced into the circulating medium by the upper and lower inlets using syringes (schematized in Figures 9A,B). Then, the system was recirculated to allow a simultaneous introduction of bacteria and Nisin to the spheroids chamber. After 48 h of continuous flow, spheroids and medium were collected and analyzed regarding the cell viability and bacterial count (both spheroid-adhered and floating) by the trypan blue and CFU count, respectively. Spheroids cultivated in the normal medium were considered as a control. Results are reported in Figure 9.

In general, results were very promising. In fact, the number of viable cells was comparable between the untreated control and infected spheroids when Nisin was added (Figure 9C). So, the cell protective while, at the same time, targeted antibacterial activity of Nisin was fully confirmed also under flow conditions. However, a slight decrease in the cell number from the starting number (4 × 10^5^) was noticed in both control (~3.4 × 10^5^) and test spheroids (~3.1 × 10^5^), which could be probably ascribed to the shear stress induced by the medium flow. In fact, undifferentiated hMSC have been demonstrated to be sensitive to mechanical stresses caused by hydrostatic pressure (Tower, 2012). However, the comparable values reported by control and Nisin treated cells suggest that Nisin did not introduce any toxic effect besides the shear stress due to the medium flow.

More importantly, the CFU count revealed that bacteria were not able to adhere to the spheroids when Nisin was introduced into the flow (Figure 9D). On the contrary, when the system was operated in the absence of Nisin, bacteria were able to adhere and colonize the spheroid. This is a crucial difference in comparison to static conditions (Figure 7B). Some explanations can be hypothesized. First of all, bacterial adhesion under static conditions is eased by the lack of shear stresses that affect, deform and remove bacterial cells from the surfaces. This was demonstrated for example by Garny et al. who showed how the shear stress applied by rotating conditions significantly reduced the biofilm adhesion, biomass and thickness in comparison to the same static conditions (Garny et al., 2008). So, hydrodynamic conditions are more difficult for biofilm development in comparison to the static cultivations. We also observed a similar trend as some bacteria colonizing the spheroid were detected in the absence of Nisin (~1.2x10^2^) but this number was lower than of that of the initial inoculum as well as the number of floating bacteria that was comparable to day 0. Second, the presence of AMPs can strongly affect the bacterial adhesion due to the membrane damaging, which could be further promoted by the shear stress. In fact, the initial bacteria adhesion is mediated by a number of cell wall proteins such as adhesins that provide anchorage to the host surface (Otto, 2013). AMPs cause severe wall damages such as pore formation due to the interaction with the membrane; this damaged wall condition leads to the failure in adhesins expression thus preventing the bacterial adhesion. Moreover, the number of floating viable bacteria was reduced in comparison to the starting number of bacteria (1 × 10^5^) thus in general confirming the Nisin antibacterial activity also under hydrodynamic conditions.

In conclusion, it can be speculated that the dynamic conditions involving the use of 3D spheroids provided better understanding of the Nisin efficacy thanks to the simultaneous combination of hMSC-shear stress-AMP effects exploited under physiologically relevant conditions. So, taking into account a possible use of a delivery system to ameliorate the AMPs performances in future studies, the complexity of the study design applied here provided the selection of the most promising peptide and the possible working concentration range.

## Conclusions

Biomaterials science offers a promising solution to solve articular cartilage repair. However, surgical implantation and the scaffold preparation accompanied with the increased bacteria resistance to antibiotics open possibilities for development of septic arthritis at increased rates. Accordingly, the use of Nisin and LL-37 antimicrobial peptides was here investigated by a novel biomimetic approach of using co-cultures of hMSC and bacteria in the presence of the antimicrobial agents. Furthermore, comparative studies in cell monolayers and in spheroid cultures indicated similar trends but also revealed the effects of the cell microenvironment, the 3D structure being more protective and inductive of cell differentiation. In addition, static and hydrodynamic culture conditions affected differently both the cells in spheroids and bacteria survival and adhesion. The hydrodynamic shear stresses were indicated as the key feature that also promoted the antibacterial activity of Nisin. These results demonstrated the importance of establishing the appropriate culture conditions in studies of novel devices and therapies that should closely resemble the physiological environment and biomimetic bioreactors may be considered as useful tools in this respect. Overall, the present work suggested that 75 μg/ml Nisin can be considered as safe and effective toward both Gram-positive and Gram-negative strains. Moreover, Nisin did not interfere with chondrogenesis thus suggesting a targeted activity toward bacteria while preserving articular cartilage healing.

## Data Availability Statement

The datasets generated for this study are available on request to the corresponding author.

## Author Contributions

ZN, AK, AS, and AC performed experiments. BO was responsible for bioreactor set-up. FG and MLe critically revised the manuscript. MLa, IL, RG, and LR designed and supervised the work. All authors agree to be accountable for the content of the work.

## Conflict of Interest

The authors declare that the research was conducted in the absence of any commercial or financial relationships that could be construed as a potential conflict of interest.
